# The Effect of Visual Attention Dispersion on Cognitive Response Time

**DOI:** 10.3390/jemr18050052

**Published:** 2025-10-10

**Authors:** Yejin Lee, Kwangtae Jung

**Affiliations:** Department of Industrial Design Engineering, Korea University of Technology and Education, Cheonan 31253, Republic of Korea; yejin3210@koreatech.ac.kr (Y.L.); ktjung@koreatech.ac.kr (K.J.)

**Keywords:** visual attention dispersion, heatmap entropy, response time, eye-tracking, Human–Machine Interface (HMI)

## Abstract

In safety-critical systems like nuclear power plants, the rapid and accurate perception of visual interface information is vital. This study investigates the relationship between visual attention dispersion measured via heatmap entropy (as a specific measure of gaze entropy) and response time during information search tasks. Sixteen participants viewed a prototype of an accident response support system and answered questions at three difficulty levels while their eye movements were tracked using Tobii Pro Glasses 2. Results showed a significant positive correlation (r = 0.595, *p* < 0.01) between heatmap entropy and response time, indicating that more dispersed attention leads to longer task completion times. This pattern held consistently across all difficulty levels. These findings suggest that heatmap entropy is a useful metric for evaluating user attention strategies and can inform interface usability assessments in high-stakes environments.

## 1. Introduction

In safety-critical environments such as nuclear power plants, airplane cockpits, sports officiating, and motor vehicle driving, operators must constantly monitor complex visual interfaces to make rapid and accurate decisions. Cognitive tasks are carried out through the information processing stages of stimulus sensation, perception, and cognition. During this process, humans detect more than 80% of information through vision [1,2,3,4]. Consequently, eye movements are closely related to the performance of cognitive tasks.

In the fields of cognitive science and human–computer interaction (HCI), understanding the user’s attention and cognitive state is crucial for system design and user experience enhancement. Eye-tracking technology is widely regarded as an effective method for achieving these objectives [5]. As a physiological measurement technique, eye-tracking records where a user is looking via the pupil, allowing researchers to objectively assess visual attention and infer underlying cognitive processes. However, pupil-based eye-tracking is not the only method to assess eye movements. Other established approaches include electrooculography (EOG), which records corneo-retinal standing potentials, and dual-Purkinje image (DPI) eye trackers, which track reflections from the cornea and lens surfaces [6].

**Figure 1:**
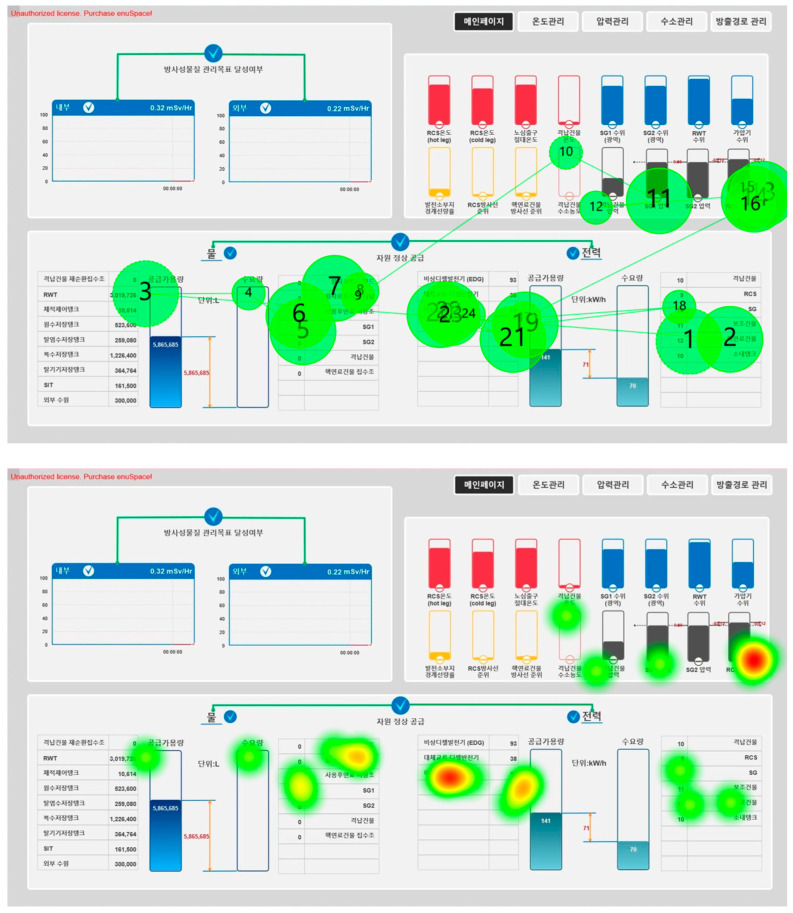
Example of a gaze plot (**top**) and a heatmap (**bottom**) representing eye movements.

Several common eye-tracking metrics have been used to analyze visual behavior, such as fixation duration, time to first fixation, fixation count, fixation ratio, and pupil size variation [7,8]. While these indicators are effective for assessing the relative importance of individual interface components, they provide limited insight into the overall spatial distribution of visual attention [9]. To address this, visualizations such as gaze plots and heatmaps are commonly employed. Gaze plots depict the temporal and spatial sequence of fixations and saccades, while heatmaps use color gradients to represent the frequency and intensity of visual attention (see Figure 1). It should be noted that such visualizations have been reinvented under different names in prior literature, including ‘recurrence plot’, ‘dot plot,’ and ‘similarity matrix’ [1], reflecting a longstanding tradition of attempts to capture the distribution of visual or cognitive activity. However, both methods are largely qualitative and lack robust quantitative descriptors [9,10].

To overcome these limitations, recent studies have applied concepts from information theory—particularly entropy measures—to quantify the complexity and dispersion of gaze behavior [11,12]. Entropy metrics based on gaze plots, such as Shannon entropy, Markov entropy, and dwell time entropy, analyze gaze transitions across predefined areas of interest (AOIs), while heatmap entropy offers a way to assess attention distribution in visually complex interfaces where AOI segmentation may not be feasible [10]. Although the Tobii Pro Lab Version 1.217 provides an overlay tool to manually anchor Areas of Interest (AOIs), applying AOI-based entropy analysis was not suitable for this study. Nuclear control room displays have well-defined panels and indicators, but the interface used in this study was a prototype accident response system containing hundreds of dynamic elements across multiple screens. Many variables (e.g., temperature, pressure, radiation levels, and flow paths) change continuously in both value and status, and their visual representations vary according to system conditions. Under such circumstances, defining stable AOIs for each element becomes impractical, as the boundaries and relevance of AOIs may shift across tasks and scenarios. Therefore, heatmap entropy was adopted to capture the global distribution of gaze without relying on predefined AOIs. According to previous studies, gaze entropy has been widely used for various research purposes, as it can quantify the degree of visual attention concentration or dispersion [13]. Alzubaidi et al. [14] analyzed gaze patterns of radiologists across experience levels when interpreting X-rays. The findings indicated that greater levels of physician experience were associated with a tendency toward lower gaze entropy. Jordan & Slater [15] applied entropy to evaluate stress effects on eye movement in VR environments. The results revealed that stress factors did not exert a significant influence on users’ gaze entropy. Gu et al. [16] used entropy to assess visual aesthetics of web interfaces. The findings indicated that while Markov entropy was not significantly associated with aesthetics, heatmap entropy was. In particular, webpages rated as more aesthetically pleasing exhibited lower levels of heatmap entropy. Hooge & Camps [17] applied it to analyze consumer gaze behavior toward brand logos in advertisements. The results demonstrated a strong positive correlation between entropy and the time to first fixation. From this, the authors suggested that advertisements providing effective gaze guidance led to more systematic eye movement patterns, which in turn corresponded to lower entropy values. Gotardi et al. [18] investigated how anxiety affects gaze behavior during driving. The study revealed that heightened anxiety was associated with increased fixation on both the roadway and the instrument panel, as well as higher levels of gaze entropy. Lee, Jung, & Lee [9] examined the correlation between gaze entropy and situational awareness during nuclear emergency simulations. The study found that gender differences significantly influenced gaze movement patterns in situation awareness, with males and females exhibiting distinct attention distribution and fixation behaviors. Jungk et al. [12] analyzed the correlation between task failure rates and gaze plot entropy across different display types—Ecological, Profilogram, and Trend displays—used for monitoring patients’ hemodynamic states. The results indicated that displays associated with lower task failure rates exhibited lower gaze entropy. This finding suggests that more intuitively designed displays promote more focused gaze behavior, which in turn is reflected in reduced entropy. Similarly, Merwe, Dijk, and Zon [3] investigated whether gaze entropy could serve as an indicator of situational awareness in flight scenarios. The study showed that specific eye movement measures, such as fixation patterns and gaze distribution, effectively reflected pilots’ levels of situation awareness during flight simulator tasks, suggesting their usefulness as objective indicators of situational awareness. Bhavsar, Srinivasan, and Srinivasan [4] utilized gaze entropy to compare the visual attention patterns of successful and unsuccessful operator groups in a chemical plant control room. As a result, the successful group exhibited lower gaze entropy than the unsuccessful group, which was interpreted as being due to their more structured eye movement patterns. Based on these findings, it was argued that entropy can be utilized as an indicator for assessing operators’ task performance levels and as a method for evaluating Human–Machine Interfaces (HMIs). Lavine et al. [19] conducted a study to explore whether eye-tracking metrics could be used as indicators of human vigilance performance, including changes in alertness and sustained attention. The results showed that changes in gaze patterns were closely linked to levels of attention and vigilance performance (alertness and sustained attention). This suggests that eye-tracking measures can serve as a useful tool for assessing human performance and fatigue.

Beyond contemporary entropy-based accounts, foundational Human Factors studies have long linked scanpath structure to mental workload. For example, Harris et al. [20] and Tole et al. [21] documented systematic changes in pilots’ scanning as workload increased. More recently, spatial-statistics approaches applied to eye movements showed that the spatial distribution of fixations is sensitive to task demand and relates to visual entropy [22,23,24,25]. Our study complements this literature by employing a spatial entropy metric—heatmap entropy—well suited to complex multi-screen interfaces where stable AOIs are difficult to define.

As demonstrated by previous studies, gaze entropy has been effectively utilized as an analytical tool across a variety of domains. Moreover, several studies have provided a robust empirical and theoretical foundation for investigating the relationship between visual attention dispersion—quantified through entropy measures—and cognitive load during information processing tasks. Table 1 summarizes the key findings of these studies.

**Table 1:** Prior studies linking gaze entropy with cognitive load.

Authors	Key Findings	Relevance to This Study
Gu et al. (2021) *Predicting webpage aesthetics with heatmap entropy* [16]	Found that higher heatmap entropy was associated with lower visual aesthetics and increased visual complexity, indicating higher cognitive load.	Supports the hypothesis that increased entropy reflects greater cognitive effort and can impair user efficiency in information-rich interfaces.
Jordan & Slater (2009) *An Analysis of Eye Scanpath Entropy in a Progressively Forming Virtual Environment* [15]	Demonstrated that stress in virtual environments increased gaze entropy, indicating attentional dispersion under cognitive strain.	Shows that psychological or cognitive stress leads to more dispersed gaze patterns, similar to what’s expected under high task difficulty in this study.
Gotardi et al. (2018) *The Influence of Anxiety on Visual Entropy of Experienced Drivers* [18]	Revealed that anxious drivers exhibited higher gaze entropy, suggesting greater visual scanning and divided attention.	Suggests a clear relationship between emotional/cognitive load and entropy, reinforcing the current study’s focus on entropy as a workload metric.
Bhavsar et al. (2017) *Quantifying situation awareness of control room operators using eye-gaze behavior* [4]	Compared successful vs. unsuccessful control room operators and found higher gaze entropy in the less successful group.	Empirically shows that higher entropy correlates with lower task performance, a key aspect of the present study’s hypothesis.
Lee et al. (2023) *Human Performance and Heat Map Entropy in System State Judgment Task using a Visual Interface Screen* [10]	In a nuclear system simulation, found a strong positive correlation between heatmap entropy and response time.	Directly precedes and justifies the current study; validates heatmap entropy as a metric for visual attention dispersion and cognitive workload.
Lavine et al. (2002) *Eye-tracking measures and human performance in a vigilance task* [19]	Used eye-tracking to show that sustained attention and reaction time vary with gaze behavior, particularly under cognitive fatigue.	Provides further evidence that response time and gaze behavior are sensitive to mental effort, aligning with this study’s objective.

In particular, previous research has demonstrated that heatmap entropy is positively associated with the time required to make judgments in visual interface tasks [10]. Specifically, our earlier study validated heatmap entropy as a predictor of task performance, showing that more dispersed gaze patterns correspond to longer judgment times and reduced accuracy. However, this work primarily examined the overall relationship between entropy and performance without considering how task complexity or cognitive demand might influence this relationship. In complex safety-critical environments, the cognitive load imposed by task difficulty can substantially alter visual search strategies. A simple trend judgment may require minimal scanning, whereas reasoning and prediction tasks necessitate broader gaze distribution and more extensive cognitive processing. These variations in task difficulty can provide a more nuanced understanding of how visual attention dispersion affects response efficiency. Therefore, the present study extends our previous work by systematically manipulating task difficulty across three levels—trend judgment, search and comparison, and reasoning and prediction—to analyze whether the entropy–response time relationship differs depending on cognitive complexity. This approach not only reaffirms heatmap entropy as a robust index of attentional dispersion but also evaluates its sensitivity to varying levels of task difficulty. In doing so, this study seeks to position entropy-based metrics as indicators of both interface usability and cognitive workload across diverse task contexts.

## 2. Methods and Materials

### 2.1. Experimental Overview

**Figure 2:**
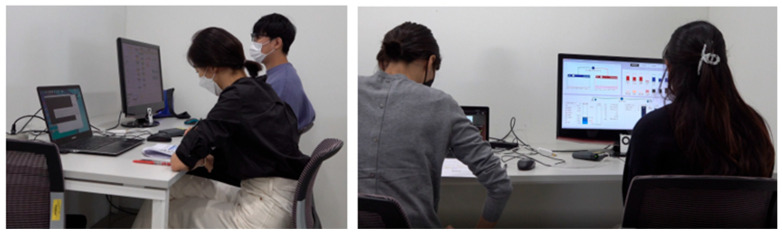
Experimental scene: the participant (subject) wearing Tobii Pro Glasses 2 is seated in front of the interface display, while the experimenter controls the scenario and records responses.

This study conducted an experiment in which participants observed the interface of a nuclear accident response support system developed using the Ecological Interface Design (EID) methodology and solved given problems based on the displayed information. The experiment was structured such that the participant viewed the interface screen and responded to questions verbally presented by the experimenter (see Figure 2).

The experimental environment consisted of a desktop computer running the accident response system, a laptop for experiment control, and an eye-tracking device. The eye-tracking system used in this study was Tobii Pro Glasses 2, a wearable device consisting of a head unit, a recording unit, and controller software. The gaze sampling frequency was 100 Hz, and corneal reflection with dark pupil tracking was employed. The average binocular accuracy under optimal conditions (illumination = 300 lux, distance = 1.5 m, gaze angle < 15°) was 0.62° (SD = 0.23), and precision was 0.05° (SD = 0.10), computed as the root mean square (RMS) of successive gaze points. The detected gaze ratio was 99% (SD = 1.7). Calibration was conducted individually for each participant by having them fixate on the center of a calibration card positioned at 0.75–1.25 m, and the calibration quality was verified via the controller software. Participants were seated comfortably without a chinrest to ensure natural posture during data collection.

The distance between the participant and the monitor was fixed at 60 cm. The experiment was conducted in a quiet laboratory setting, free from external disturbances. Prior to participation, all participants were provided with a full explanation of the experimental objectives and procedures and signed a consent form voluntarily.

A total of 16 undergraduate engineering students (8 males and 8 females) participated in the experiment. None of the participants had prior experience with nuclear accident response systems. The average age of the participants was 22.3 years (SD = 1.5). All participants had normal or corrected-to-normal vision. Before proceeding to the main experiment, each participant underwent training to understand the system’s structure, variables, and their behavior. Only those who correctly answered all 10 practice questions presented by the experimenter were allowed to proceed.

A total of 21 simulation scenarios were developed, each lasting approximately 5 min. The experimental scenarios were randomly selected to minimize order effects and participant bias. During each scenario, participants responded to questions based on the information displayed on the screen. Both response time and eye movement data were recorded. Response time was defined as the duration from the moment the question was presented by the experimenter to the moment the participant provided an answer. Each observation corresponds to a trial, defined as one participant answering one question within a simulation scenario. For each trial, we computed one heatmap entropy value from gaze data between question onset and the participant’s verbal answer, and one response time measured from the experimenter’s verbal prompt to the participant’s verbal response. Only trials with complete gaze and response-time recordings were included; trials with tracking loss or missing responses were excluded. After the experiment, participants completed a post-task questionnaire to rate the cognitive difficulty of each task type using a 7-point Likert scale.

### 2.2. Prototype Interface Design

**Figure 3:**
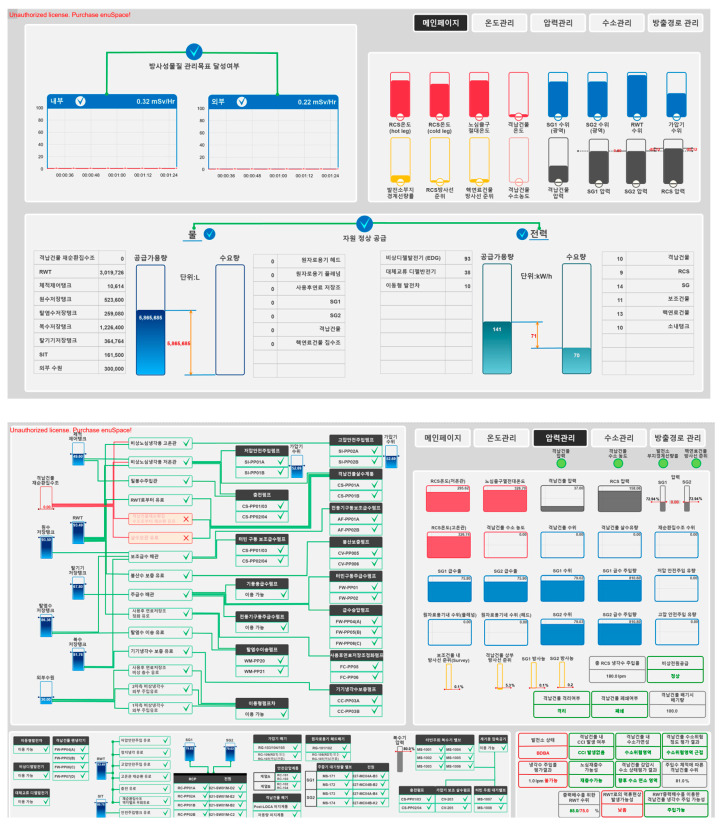
Prototype interface: main screen (**top**) and pressure control screen (**bottom**).

The system used in the experiment was a simulation-based interface developed based on the principles of EID, a framework that visualizes the relationships among work domain constraints and various levels of complex information, enabling operators to understand the system state and solve problems effectively [26]. The interface had a two-tier structure, composed of a main screen and four subordinate screens (see Figure 3).

(1) Main Screen

The main screen was divided into four quadrants, each providing different types of information relevant to system monitoring and control. The upper left area displays the achievement status of radiation safety goals. When both internal and external radiation levels remain below the designated thresholds, a green checkbox is shown; otherwise, a red “X” appears. Graphical indicators also represent the current radiation values for both internal and external areas. The upper right area provides an overview of key system variables—such as temperature, pressure, water level, and radiation levels—using color-coded gauges and trend indicators. This section also includes navigation buttons for accessing the four subordinate control screens. The lower left area visualizes the supply and demand of cooling water. It shows the available supply from tanks and external sources, the demand from subsystems, and the supply–demand difference using gauges and bar charts. Finally, the lower right area presents the electric power supply and demand. It displays the available power from each generator, the power demand from various facilities, and the overall power balance in a visually intuitive format.

(2) Subordinate Screens

The four subordinate screens—temperature, pressure, hydrogen, and release path control—share a consistent layout composed of four regions. The upper left area displays flow path availability. The status of each path (available, unavailable, or in-use) is represented using colored lines and icons, determined by the condition of corresponding water sources and driving power. The upper right area includes navigation buttons for screen transitions and status indicators. Green denotes normal operating conditions, while red indicates abnormal states. Current values of relevant variables are also displayed. In the lower left area, the availability of system devices is visualized using colored boxes and connection lines—green for available devices and red for unavailable ones. The lower right area shows the numeric values of critical variables. Values within the normal range are displayed in green, while out-of-range values are shown in red.

### 2.3. Task Design

To account for variations in cognitive workload and visual search demand, three types of problem-solving tasks were developed. Each task type was designed to represent a distinct level of cognitive complexity and attentional demand during interaction with the interface. A total of 24 experimental questions were created, with 8 questions per task type, all designed to have equivalent difficulty across conditions. The task types are shown in Table 2.

These three task types were deliberately structured to systematically distinguish and analyze the participants’ cognitive processing and visual attention dispersion during information search. Sixteen participants completed 15 trials each (balanced across the three task types), yielding 16 × 15 = 240 valid trials analyzed in the correlation between heatmap entropy and response time. The 24 questions (8 per task type) served as a pool from which each participant’s 15 trials were sampled, and 21 simulation scenarios provided the dynamic context; assignment was randomized across sessions.

### 2.4. Participant Training

To ensure proper task performance and measurement accuracy, participants underwent a two-stage training process:

(1) System and Variable Orientation

Participants received detailed explanations of the system interface layout and variable types. This included how variables such as water level, pressure, and power were displayed using color codes, gauges, and trends. Simulation videos were used to aid in understanding how values were visualized and interpreted on each screen.

(2) Scenario-Based Practice

Participants observed simulation videos while the experimenter verbally posed example questions. The training continued until the participant was able to correctly answer 10 consecutive practice questions. Only after passing this criterion did participants proceed to the formal experiment.

### 2.5. Heatmap Entropy

A heatmap is a visualization index that represents the intensity of participants’ visual attention. This study employed heatmap entropy as an index of gaze movement. In this study, the heatmap was constructed by spatially sampling fixation points across the entire screen using a Gaussian kernel density estimation method. The screen was divided into a grid of 1280 × 1024 pixels (corresponding to the resolution of the display), and each fixation was mapped to the grid.

**Figure 4:**
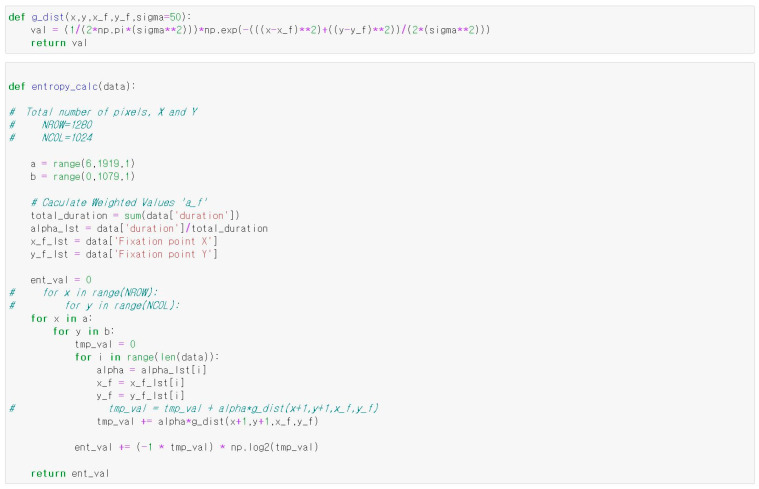
Python code for heatmap entropy calculation.

We used a kernel standard deviation of σ = 30 pixels (≈0.5° of visual angle), corresponding to the effective resolution of the Tobii Pro Glasses 2. Fixation detection was conducted using the I-DT (dispersion-threshold) algorithm, with a minimum fixation duration of 100 ms and a dispersion threshold of 1°. A fixation was defined as gaze maintained for at least 60 ms, whereas a saccade was identified when the gaze shifted more than 100° within 1 s. These thresholds align with previous eye-tracking literature. To ensure reproducibility, the full Python 3.11 implementation used to calculate heatmap entropy is provided in Figure 4.

A Gaussian distribution was applied to each fixation, and the overlapping distributions were summed to form a continuous probability distribution across the screen. This probabilistic distribution was then used to calculate the heatmap entropy.

Because the heatmap has the form of a Gaussian distribution from the point where the gaze stays the longest to the point where the gaze stays the least, heatmap entropy is calculated using Gaussian distribution. Assuming that the gaze follows a Gaussian distribution centered on a specific pixel (x_f_, y_f_), the continuous probability distribution of the gaze forming the heatmap is expressed by the following Equation (1) [10].

(1) $$f_{XY}\left(x,y\right)=\frac{1}{2\pi \sigma^{2}}exp-(\frac{{\left(x-x_{f}\right)}^{2}+{\left(y-y_{f}\right)}^{2}}{{2\sigma }^{2}})$$

where σ denotes the standard deviation, representing the range of pixels that a user can perceive when viewing the screen. If multiple fixation distributions are formed on the screen, it is necessary to assign weight to the fixation distribution and express it as one continuous probability distribution, as follows:

(2) $${\tilde{f}}_{XY}(x,y)=\sum_{f=1}^{f_{num}}d_{f}\times \frac{1}{{2\pi \sigma }^{2}}exp-(\frac{(x-x_{f}{)}^{2}+(y-y_{f}{)}^{2}}{{2\sigma }^{2}})$$

where f_num_ is the number of fixations and d_f_ is the weight of each fixation distribution. The heatmap entropy can be calculated using this joint probability distribution function [10].

(3) $$H=-\sum_{xy}\tilde{f_{xy}}(x,y)\cdot log{\tilde{f}}_{xy}(x,y)$$

The heatmap entropy was calculated using Python 3.11. The following Python code was used to calculate the heatmap entropy.

## 3. Results

### 3.1. Relationship Between Heatmap Entropy and Judgment Time

**Figure 5:**
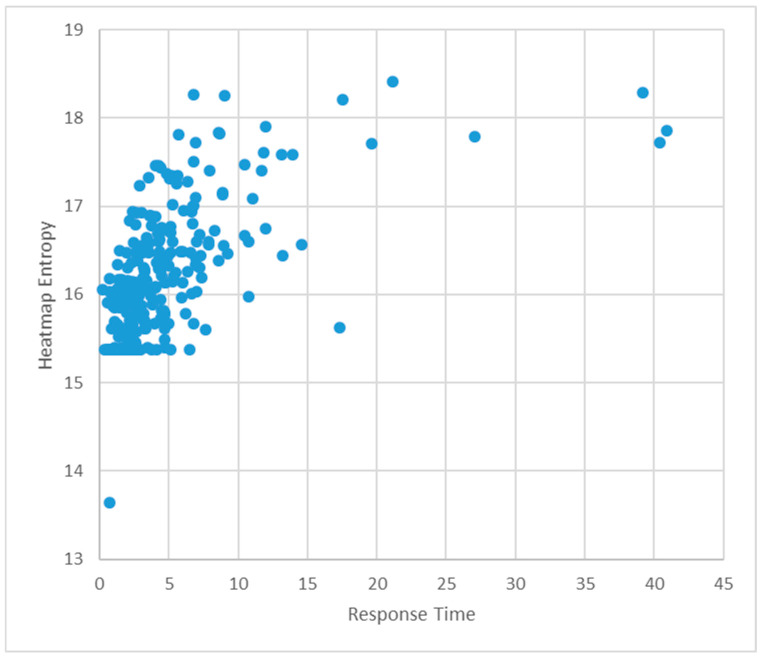
Scatter plot between response time (seconds) and heatmap entropy.

To investigate the overall relationship between heatmap entropy and participants’ judgment time during information search, a Pearson correlation analysis was performed using the full dataset (N = 240). Response time (R_time) was defined as the duration from the moment the question was presented by the experimenter until the participant provided an answer. As illustrated in Figure 5, the scatter plot revealed a clear linear trend in which response time increased in proportion to heatmap entropy, suggesting that greater visual attention dispersion corresponds with longer response times.

The correlation coefficient was r = 0.595 (*p* < 0.01), indicating a statistically significant and moderately strong positive correlation between the two variables (see Table 3). This means that as participants’ gaze became more dispersed across the interface, they required more time to locate and process relevant information. This trend implies that heatmap entropy may serve as a robust indicator of the cognitive demand imposed by the interface layout.

To further quantify the predictive relationship between heatmap entropy (HE) and response time (RT), a simple linear regression analysis was conducted. The resulting regression equation was:

RT = –64.76 + 4.28 × HE

The regression model was statistically significant (*p* < 0.01), with an R^2^ value of 0.354, indicating that approximately 35.4% of the variance in response time can be explained by variations in heatmap entropy.

Although the scatter plot displays clustered regions, these clusters primarily reflect heterogeneity by task type (Types 1–3), which we analyze separately in Section 3.3. We therefore report the linear fit as a parsimonious, descriptive summary of the overall monotonic association between heatmap entropy (HE) and response time (R_time), and note that simple higher-order specifications and smooth fits did not materially change this conclusion.

### 3.2. Difficulty Analysis by Task Type

**Figure 6:**
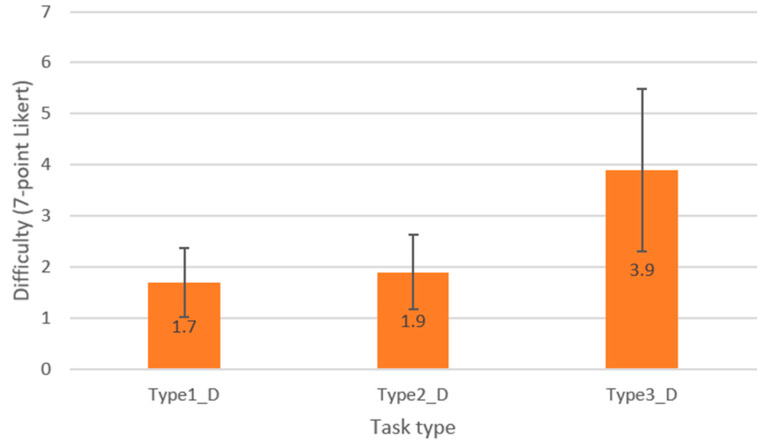
Mean difficulty scores by task type (*y*-axis: Difficulty, 7-point Likert).

To analyze the changes in response time according to visual attention dispersion during the information search process on a visual interface screen, a preliminary analysis was conducted to determine whether there were significant differences in perceived task difficulty across the three task types. Task difficulty was evaluated using a 7-point Likert scale, and Figure 6 presents the average difficulty ratings for each task type. The analysis revealed that task type 3 was perceived as the most difficult, followed by Type 2, and then Type 1. A one-way ANOVA confirmed that these differences were statistically significant at the 0.05 significance level (*p* < 0.05). These findings suggest that the problems used in the experiment were appropriately designed to reflect distinct levels of difficulty. Based on this, a further analysis was conducted to examine whether a significant correlation between response time and heatmap entropy also exists under conditions of varying task difficulty.

### 3.3. Variation in Response Time According to Heatmap Entropy by Task Type

Beyond reporting directional differences, we summarize the descriptive statistics of heatmap entropy (HE) and response time (R_time) by task type. Mean (SD) HE values were Type 1 = [16.46 ± 0.92], Type 2 = [16.05 ± 0.5], and Type 3 = [16.18 ± 0.65]; corresponding R_time values were Type 1 = [6.89 ± 4.79], Type 2 = [3.31 ± 1.78], and Type 3 = [4.02 ± 3.29]. In this study, considering that response time and heatmap entropy may vary depending on task difficulty, the relationship between these two variables was analyzed by task types.

For task type 1, which was rated as the least difficult, an initial analysis was conducted to determine whether heatmap entropy and response time differed significantly by gender. The results showed no statistically significant differences in heatmap entropy (*p* > 0.05) or response time (*p* > 0.05) by gender at the 0.05 significance level. Therefore, the correlation analysis between heatmap entropy and response time was performed without separating participants by gender.

**Figure 7:**
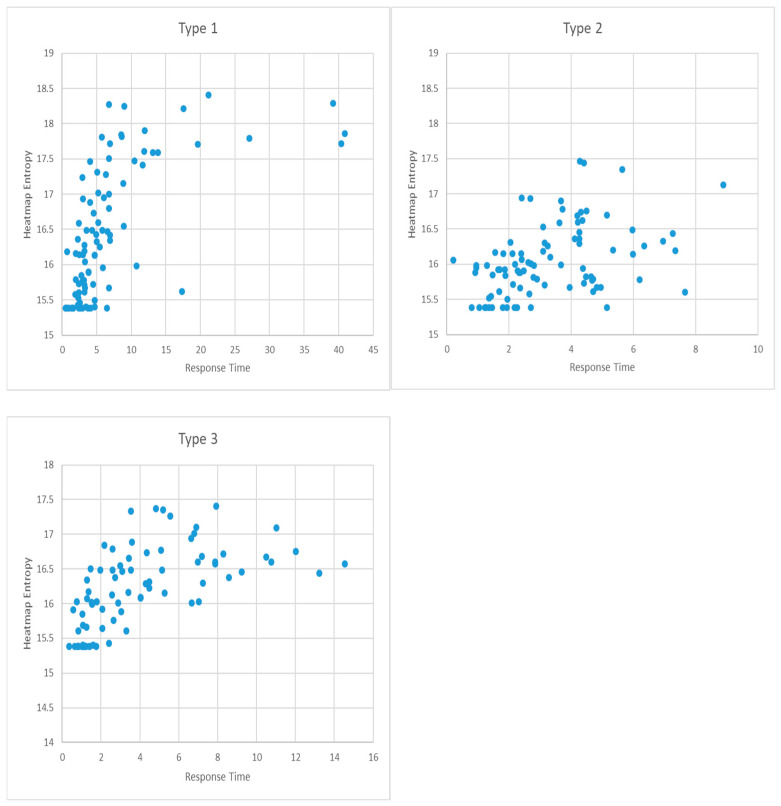
Scatter plots of response time and heatmap entropy by task type (**Top Left**: Type 1, **Top Right**: Type 2, **Bottom**: Type 3).

As shown in Figure 7, which presents the scatter plot between response time and heatmap entropy, a general tendency is observed whereby heatmap entropy increases as response time increases. To quantitatively examine this linear relationship, a correlation analysis was conducted. The results revealed a statistically significant correlation between the two variables at the 0.01 level (*p* < 0.01), with a correlation coefficient of 0.614, indicating a strong positive relationship.

Given the strong correlation between response time (RT) and heatmap entropy (HE), a regression analysis was performed to identify the functional relationship between the two variables. The resulting regression equation was as follows, and it was found to be statistically significant at the 0.01 level (*p* < 0.01):

RT = −78.494 + 5.188 × HE

The regression model was statistically significant (*p* < 0.01), with an R^2^ value of 0.377, indicating that 37.7% of variance in RT is explained by HE. These results indicate a strong positive correlation, suggesting that even relatively simple tasks require more time as attention becomes more dispersed, perhaps due to inadequate guidance of attention in interface layout.

The same analysis was conducted for task type 2. As with Type 1, no statistically significant gender differences were found for heatmap entropy (*p* > 0.05) or response time (*p* > 0.05); therefore, the correlation analysis was also conducted without separating by gender.

The scatter plot shown in Figure 5 also indicates a tendency for heatmap entropy to increase as response time increases. The correlation analysis revealed a statistically significant relationship at the 0.01 level (*p* < 0.01), with a correlation coefficient of 0.422, indicating a moderately strong correlation. Based on the regression analysis, the following equation was obtained:

RT = −20.697 + 1.496 × HE

The regression model was statistically significant (*p* < 0.01), with an R^2^ value of 0.178, suggesting a moderate association between HE and RT. Here, the increase in entropy is accompanied by a moderate rise in response time, reflecting the need to scan and compare multiple elements simultaneously, increasing the visual search space.

For task type 3, the same analytical procedure was applied. As with Types 1 and 2, neither heatmap entropy (*p* > 0.05) nor response time (*p* > 0.05) showed statistically significant gender differences. Therefore, the analysis was again conducted without gender separation.

In the scatter plot shown in Figure 5, there is a clear tendency for heatmap entropy to increase as response time increases. A correlation analysis confirmed a statistically significant relationship at the 0.01 level (*p* < 0.01), with a correlation coefficient of 0.585, indicating a strong positive correlation. The regression analysis yielded the following equation:

RT = −44.326 + 2.987 × HE

The regression model was statistically significant (*p* < 0.01), with an R^2^ value of 0.342, again indicating a strong relationship. This task type involves the most complex cognitive reasoning, and the results suggest that increased gaze dispersion during inference and mental simulation significantly slows down response time, reinforcing the connection between entropy and cognitive effort.

Across all task types, a consistent pattern was observed: higher heatmap entropy values were associated with longer response times, regardless of the task’s cognitive complexity level. However, the strength of the correlation and the slope of the regression line varied, indicating that the degree to which attentional dispersion influences response time is task-dependent.

These findings support the conclusion that heatmap entropy is a valid and sensitive metric for quantifying attentional dispersion and cognitive effort, especially in visually complex or time-critical interface environments. It also demonstrates that entropy-based measures can be meaningfully incorporated into the usability evaluation of system interfaces, particularly in safety-critical contexts such as nuclear control systems.

## 4. Conclusions

This study demonstrates that, on a complex nuclear accident-response display, greater dispersion of visual attention measured by heatmap entropy corresponds to longer response times. The relationship of heatmap entropy and response time held across three levels of task difficulty, with task-dependent slopes that reflect differing cognitive demands. Accordingly, HE provides a workload-sensitive metric that can inform usability assessment and design decisions in safety-critical interfaces.

To this end, an experiment was conducted using a prototype of an accident response support system for a nuclear power plant. The experiment measured participants’ response times and gaze data while they solved information search problems with varying levels of difficulty. In general, to quantify visual attention dispersion based on gaze movement data, information-theoretic metrics such as Shannon entropy, Markov entropy, and heatmap entropy can be used. Among these, Shannon and Markov entropy require predefined Areas of Interest (AOIs) based on specific screen elements. However, in complex interfaces—such as the one used in this study, which includes many visual components—defining fixed AOIs is not suitable. Therefore, this study employed heatmap entropy, which reflects the spatial distribution of gaze across the entire screen without requiring AOI segmentation.

When analyzing the relationship between heatmap entropy and response time across all tasks without considering task difficulty, the results showed a statistically significant positive correlation at the 0.01 significance level, with a relatively high correlation coefficient of 0.595. Further analysis was conducted by task type (reflecting different difficulty levels), and the results consistently revealed significant positive correlations between response time and heatmap entropy across all task types. These findings indicate that greater dispersion of visual attention is associated with longer response times in problem-solving. In other words, the increase in heatmap entropy with longer response times suggests that participants distributed their gaze more broadly across the screen as they explored more visual elements to reach a decision.

The present findings confirm and extend our earlier results [10], which demonstrated a general positive correlation between heatmap entropy and judgment time. While the previous study established entropy as a predictor of overall task performance, the current study advances this understanding by showing that the strength and slope of the entropy–response time relationship vary systematically with task difficulty. Specifically, simpler tasks (trend judgments) showed a stronger entropy–time correlation, whereas more complex tasks (reasoning and prediction) revealed a different slope, reflecting distinct cognitive demands.

First, the analysis across all tasks demonstrated a strong positive correlation between heatmap entropy and response time, confirming that greater attentional dispersion consistently leads to slower information processing. Second, when considering task difficulty, the correlation strength and regression slope varied by task type, with simpler tasks producing stronger associations and complex reasoning tasks showing distinct patterns. Third, the regression equations indicated that entropy values can quantitatively predict the degree of response delay under varying cognitive demands. Taken together, these three findings validate our stated research goal—that heatmap entropy can serve as a quantitative indicator for evaluating information load.

Compared to transition matrix entropy, which requires predefined Areas of Interest (AOIs) and is highly sensitive to AOI definitions, heatmap entropy can be applied without segmentation, making it more robust in complex multi-element displays. Spatial distribution indices capture only the spread of fixations but do not incorporate the probabilistic structure of gaze distribution. In contrast, heatmap entropy models the full distribution of attention across the display, providing a unified measure sensitive to both concentration and dispersion. This characteristic makes it especially suitable for dynamic, safety-critical interfaces where AOI boundaries are unstable. Thus, our findings extend previous scanpath entropy and workload studies by demonstrating that heatmap entropy offers a practical and theoretically grounded approach to quantify attentional dispersion in such environments.

This extension highlights two important contributions. First, heatmap entropy is not merely a static indicator of gaze dispersion; rather, it is sensitive to the level of cognitive complexity inherent in the task. Second, by incorporating task difficulty into the analysis, the study demonstrates that entropy can serve as a diagnostic metric for identifying conditions under which visual interface design may hinder efficient information search. Taken together, these results suggest that entropy-based measures can move beyond performance prediction to function as dynamic indicators of cognitive workload, offering practical utility in evaluating and optimizing interface design in safety-critical systems.

This phenomenon can be interpreted as a sign that users had difficulty quickly locating necessary information, implying potential design inadequacies in the interface. In other words, inefficient interface design leads to an increased frequency and dispersion of gaze, resulting in higher heatmap entropy and longer response times. Therefore, heatmap entropy can serve not only as a predictive indicator of response time but also as a quantitative metric for evaluating interface design quality.

The results of this study offer valuable implications for the design and evaluation of visual interfaces in high-stakes decision-making environments such as nuclear power plants. The strong correlation between heatmap entropy and response time suggests that gaze-based metrics can be effectively used to identify inefficiencies in interface layout and structure. By analyzing heatmap entropy as an indicator of attentional dispersion, it becomes possible to objectively detect cognitive overload or challenges in locating key information during specific tasks. Therefore, actively incorporating visual attention dispersion metrics—such as heatmap entropy—into interface design and usability evaluations can lead to the development of more intuitive and cognitively efficient user interfaces that better align with users’ natural information-seeking strategies. Therefore, actively incorporating visual attention dispersion metrics—such as heatmap entropy—into interface design and usability evaluations can lead to the development of more intuitive and cognitively efficient user interfaces that better align with users’ natural information-seeking strategies.

In future research, it would be valuable to extend this approach to real-time monitoring systems and dynamic interfaces, where attention patterns may vary in response to changing operational contexts. Incorporating physiological data such as heart rate variability, EEG, or pupil dilation alongside gaze metrics could provide a more comprehensive understanding of the user’s cognitive state. Furthermore, longitudinal studies examining how user expertise or training impacts heatmap entropy and response performance over time would help validate the robustness of entropy-based indicators. Potential applications of this research span a wide range of domains, including nuclear operations, air traffic control, medical diagnostics, military command interfaces, and even consumer-grade systems such as automotive dashboards and smart home control panels. In all these domains, interface complexity and time-critical decision-making are central concerns, and entropy-based visual attention analysis could be instrumental in optimizing usability, reducing cognitive load, and enhancing operational safety.

## Figures and Tables

**Table 2:** Task types and example questions.

Task Type	Description	Example Question
Type 1: Trend Judgment Tasks	Determine the directional change (increase or decrease) of a single variable over time.	“How is the SG1 water level changing?”
Type 2: Search and Comparison Tasks	Identify a variable that satisfies a specific condition by searching and comparing multiple variables.	“Which facility has the highest electricity demand?”
Type 3: Reasoning and Prediction Tasks	Predict system behavior or outcomes under a hypothetical condition or manipulation.	“If both desalinated water transfer pumps are stopped, which flow path will become unavailable?”

**Table 3:** Correlation analysis between heatmap entropy and judgment time.

		Response Time
Heatmap entropy	Pearson Correlation	0.595 **
	Sig. (2-tailed)	0.000
	N	240

** The coefficient is significant at the 0.01 level (2-tailed).

## Data Availability

Dataset available on request from the authors.
